# Peripheral Blood T‐Cell Receptor Repertoire Diversity as a Potential Biomarker in the Diagnosis and Treatment Evaluation of Colorectal and Lung Cancers: A Prospective Observational Study

**DOI:** 10.1002/cam4.70937

**Published:** 2025-05-19

**Authors:** Jilong Ma, Yuanbiao Wang, Zhixin Zhang, Xinyi Cai, Xudong Xiang, Yan Chen, Fengqiong Sun, Jian Dong

**Affiliations:** ^1^ Key Laboratory of Cell Therapy Technology Transformation Medicine of Yunnan Province, the Han Weidong Expert Workstation of Yunnan Province, Yunnan Provincial Engineering Research Centre of Cell Therapy and Quality Control System, the Third Affiliated Hospital of Kunming Medical University, Yunnan Cancer Hospital Kunming Yunnan China; ^2^ Department of Technology Chengdu ExAb Biotechnology, LTD Chengdu Sichuan China; ^3^ Department of Colorectal Surgery The Third Affiliated Hospital of Kunming Medical University, Yunnan Cancer Hospital Kunming Yunnan China; ^4^ Department of Thoracic Surgery II The Third Affiliated Hospital of Kunming Medical University, Yunnan Cancer Hospital Kunming Yunnan China

**Keywords:** colorectal cancer, neoadjuvant therapy, nonsmall‐cell lung cancer, prospective observational study, TCR repertoire diversity

## Abstract

**Background:**

T‐cell receptor (TCR) diversity 50 (D50) values could assess peripheral blood (PB) TCR diversity and immunity. This study aimed to evaluate the potential D50 value in the diagnosis and treatment evaluation of colorectal cancer (CRC) and nonsmall‐cell lung cancer (NSCLC).

**Methods:**

This prospective observational study enrolled patients with CRC, benign colorectal disease (BCD), NSCLC, or benign nodule controls (BNC) and healthy donors (HD) at Yunnan Cancer Hospital between January 2021 and June 2022. PB specimens were used for TCRβ sequencing, and D50 was calculated and compared within different groups. The area under the curve (AUC) was used to evaluate the diagnostic performance of D50 in CRC and NSCLC.

**Results:**

A total of 114 HD and 115 CRC, 31 BCD, 67 NSCLC, and 25 BNC patients were enrolled. Both CRC and NSCLC patients exhibited significantly lower D50 compared with HDs (*p* < 0.001), whereas BCD and BNC patients showed a modest decrease in TCR diversity (*p* < 0.05). NSCLC patients with lymph node metastases had markedly lower D50 than those without lymph node metastasis (0.05 vs. 0.11, *p* < 0.01). Higher D50 was found in CRC and NSCLC patients with normal carcinoembryonic antigen (CEA) levels (*p* < 0.05). The potential of D50 value for early detection of CRC and NSCLC was demonstrated, with an area under the receiver operating characteristic curve (AUC) of 0.736 for CRC (sensitivity: 71.30%, specificity: 68.42%) and 0.768 for NSCLC (sensitivity: 83.58%, specificity: 60.53%). Significant differences in D50 values were observed between patients with tumor regression grade (TRG) 0–1 and those with TRG 2–3 (*p* = 0.027), with an AUC of 0.731 (sensitivity: 68.75%, specificity: 76.92%).

**Conclusion:**

These findings suggest that the PB TCR D50 values may have significant clinical value in cancer diagnosis and in evaluating the efficacy of neoadjuvant therapies.

## 1 Introduction

Colorectal cancer (CRC) is the second leading cause of cancer‐related death and causes almost 900,000 deaths annually [1]. According to the United States Preventive Services Task Force (USPSTF), colorectal cancer screening is recommended for individuals at risk beginning at 45 years of age [2, 3]. Nonsmall‐cell lung cancer (NSCLC) is the most common lung cancer [4]; it has a high prevalence and mortality, and its prevalence is expected to increase further [5]. Immunity plays an important role in the development of CRC and NSCLC. T lymphocytes are part of the adaptive immune system and play crucial roles in eliminating pathogens and tumor immunity, and the T‐cell receptors (TCRs) located on the T‐cell surface are key to selectively recognizing tumor antigens and killing tumor cells [6]. During the initial development phases, T lymphocytes produce TCRs that recognize various antigens by generating diverse V‐J gene combinations in the complementarity‐determining region 3 (CDR3) of the TCRβ, thereby establishing TCR repertoire diversity [7]. The diversity of the TCR repertoire is a direct reflection of the diversity found in cellular immunity, and the lack of TCR diversity is indicative of immune system dysfunction.

The variation of TCR diversity is not only the foundation of human immune function but also a potential biomarker for certain diseases such as cancers and viral infections [8, 9]. Changes in TCR repertoires in the peripheral blood (PB) and tumor‐infiltrating lymphocytes (TILs) have been observed in patients with tumors [10]. Currently, primary methods for diagnosing solid cancers involve pathological biopsy and imaging tests [11, 12, 13]. However, a pathological biopsy is an invasive procedure, while imaging tests, including computed tomography and magnetic resonance, can potentially miss small nodules and tumors. Meanwhile, the TCR diversity evaluated using high‐throughput sequencing techniques provides a noninvasive approach to cancer detection. The diversity of TCRβ CDR3 genes in T lymphocytes enables the calculation of TCR diversity 50 (D50) values, providing a quantitative biomarker for immunity and disease severity assessment [14]. Moreover, the diversity of the TCR repertoire has been associated with the prognosis of certain diseases. PB TCR diversity analysis can be a new and noninvasive approach for diagnosing tumors.

Immune function is crucial for the inhibition of tumor initiation and metastasis [15]. Immunotherapy is widely adopted in clinical practice for cancer patients. Neoadjuvant immunotherapy is a new strategy with great potential, and several studies have revealed its effectiveness in CRC [16] and NSCLC [17]; however, currently, the neoadjuvant therapy efficacy can only be evaluated clinically through the postoperative pathological tumor regression grade (TRG) [18]. Therefore, novel biomarkers are needed to predict preoperative neoadjuvant therapy efficacy. Although tumor markers, including carcinoembryonic antigen (CEA), are reliable indicators of disease progression in CRC and NSCLC [19, 20, 21], due to the non‐specific nature of these biomarkers, they cannot be used solely in clinical practice. Therefore, it is necessary to explore novel markers for cancer progression prediction, and PB TCR diversity may be a reliable option.

Therefore, it was hypothesized that the differences in amplified fragments lead to changes in the TCR diversity of cancer patients and that the differences may be used to distinguish between individuals with versus without cancer as a diagnostic method. It was also hypothesized that TCR diversity can predict the response to neoadjuvant treatment in patients with CRC. This study aimed to analyze and evaluate the clinical application values for TCR diversity in healthy controls and patients with CRC and NSCLC.

## 2 Materials and Methods

### 2.1 Study Design and Patients

This prospective observational study enrolled patients with CRC, benign colorectal disease (BCD), NSCLC, benign nodule controls (BNC), and healthy donors (HD) at Yunnan Cancer Hospital (Kunming City, Yunnan Province, China) between January 2021 and June 2022. HD was defined as individuals without any history of benign or malignant tumors. The inclusion criteria for the CRC group were (1) patients diagnosed with CRC through colonoscopy histopathology or postoperative pathology and (2) no prior antitumor treatment before blood sampling. The inclusion criteria for the BCD group were (1) patients diagnosed with BCD through colonoscopy histopathology or postoperative pathology and (2) no prior antitumor treatment before blood sampling. The inclusion criteria for the NSCLC group were (1) patients diagnosed with NSCLC through preoperative histopathology or postoperative pathology and (2) no prior antitumor treatment before blood sampling. The inclusion criteria for the BNC group were (1) patients diagnosed with BNC through preoperative histopathology or postoperative pathology and (2) no prior antitumor treatment before blood sampling. The exclusion criteria for all participants were (1) autoimmune diseases, HIV, tuberculosis, or active hepatitis; (2) in the acute stage of infection; (3) undergoing treatment with hormones or immunosuppressive agents; or (4) received organ transplants. This study was approved by the Medical Ethics Committee of Yunnan Cancer Hospital (KYLX2022201). Before sample collection, all participants completed an informed consent form.

### 2.2 RNA Extraction

A 1‐mL venous blood sample was collected in an EDTA anticoagulant tube containing 5 mL of TRIzol reagent and allowed to stand at 25°C for 5 min to ensure complete cell lysis. The RNA extraction was performed using the standard Trizol method (Supplementary Methods).

### 2.3 Polymerase Chain Reaction (PCR) Amplification of the TCRβ Gene

The initial phase of PCR amplification for the TCRβ gene was performed with a QIAGEN One‐Step RT‐PCR Kit (QIAGEN, Cat. 210,212 QIAGEN, Venlo, The Netherlands) and a template composed of RNA isolated from PB mononuclear cells (PBMCs). The conditions of the first round of PCR were as follows: one cycle at 50°C for 30 min and 95°C for 15 min, followed by 20 cycles at 95°C for 30 s, 58°C for 30 s, and 72°C for 30 s. A second round of PCR amplification was performed on the TCRβ gene employing the previous PCR products as a template. The second round of PCR was conducted as follows: one cycle at 95°C for 10 min, followed by 20 cycles at 95°C for 30 s, 58°C for 30 s, and 72°C for 30 s. The PCR product was purified using a DNA Fragment Sorting and Purification Kit (BioMagbeads, Wuxi, China, Cat. BMSX).

### 2.4 Sequencing of the TCRβ Gene

A ThermoFisher Ion Plus Fragment Library Kit (Cat. 4471252 ThermoFisher Scientific, Waltham, MA, USA), Ion Xpress Barcode Adapters 1–16 Kit (Cat. 4471250 Thermo Fisher Scientific, Waltham, MA, USA), and Agencourt AMPure XP (Cat. A63881 Thermo Fisher Scientific, Waltham, MA, USA) were used to construct a sequencing library. Automated template preparation was performed using the Thermo Fisher Ion Chef System and TCRβ gene sequencing using the Thermo Fisher Ion S5 system. Sequencing data were exported and separated into individual FAST‐All (FASTA) files for each sample based on their respective barcodes. The local IgBLAST program was used to analyze the FASTA files.

### 2.5 D50 Values

TCRβ repertoire diversity was quantified using the D50 values. The D50 values were determined as the proportion of dominant TCRβ CDR3 clone types in each sample, accounting for 50% of the total TCRβ sequences [14]. The calculation formula was as follows: *N* represents the total count of TCRβ sequences present in the sample, whereas C represents the count of distinct TCRβ CDR3 amino acid sequence types. The functional TCRβ sequences corresponding to each distinct CDR3 sequence were assigned as *N*1, *N*2, …, *NC*, and arranged in descending order of size as *N*1 ≥ *N*2 ≥ … ≥ *NC* − 1 ≥ *NC*. When (*N*1 + *N*2 + … + *NH* − 1) ≤ 0.5 × *N* and (*N*1 + *N*2 + … + *NH*) ≥ 0.5 × *N*, and D50 represents the ratio of *H* to *C*.

### 2.6 Shannon Entropy

The calculation formula for Shannon entropy is as follows:

$$H^{\prime }=-\sum_{i=1}^{S}\mathrm{pi}\ln\mathrm{pi},$$

where *S* represents the total number of unique T‐cell receptor (TCR) clonotypes in the sample, and *pi* is the relative abundance of the *i*th clonotype.

Range: The value of *H'* satisfies *H'* ≥ 0. A higher value indicates greater diversity. When all clonotypes are evenly distributed, it reaches the maximum value *H*_max'_ = ln *S* [22].

### 2.7 Simpson Index

The calculation formula for the Simpson index is as follows:

$$D=1-\sum_{i=1}^{S}p_{i}^{2},$$

where *S* represents the total number of unique T‐cell receptor (TCR) clonotypes in the sample, and *p*_*i*_ is the relative abundance of the *i*th clonotype.

Range: The value of *D* satisfies 0 ≤ *D* < 1. A higher value indicates fewer dominant clonotypes and higher diversity. The original Simpson index ($\lambda =\sum p_{i}^{2}$) is often converted to 1 − *λ* or *λ*_1_ to avoid confusion [23].

### 2.8 The Detection of Serum Tumor Markers

Peripheral venous blood samples (5 mL) were obtained from each patient diagnosed with CRC before treatment and after an overnight fast. The samples were collected in EDTA anticoagulant tubes and centrifuged at 1500×g for 10 min. The resulting supernatant was separated and analyzed for CEA levels using an electrochemiluminescence immunoassay (ECLIA).

### 2.9 Statistical Analysis

The statistical analysis was performed using SPSS 26.0 (IBM Corp., Armonk, NY, USA). The continuous data were expressed as the median (interquartile range) or mean ± standard deviation. Wilcoxon test or Student's *t*‐test was used to assess disparities between two groups, whereas the Kruskal–Wallis test or analysis of variance (ANOVA) was used to evaluate disparities among multiple groups. The chi‐squared test was performed for categorical data to evaluate the differences among the groups. The level of statistical significance was set at *p* < 0.05. The diagnostic potential of the D50 value for early detection and its predictive ability for neoadjuvant therapy efficacy was assessed using ROC curves.

## 3 Results

### 3.1 Demographic Characteristics

A total of 114 HDs, 115 CRC patients, 31 BCD patients, 67 NSCLC patients, and 25 BNC patients were collected (Figure S1). The average age for HD, CRC, BCD, NSCLC, and BNC was 52.18 ± 8.39, 56.48 ± 11.25, 51.03 ± 13.73, 54.01 ± 10.00, and 45.40 ± 10.47 years old; the males constituted 40.4%, 60.0%,61.3%, 40.3%, and 40.0% in every group, respectively, and smokers represented 28.1%, 32.2%, 25.8%, 26.9%, and 28.0%, respectively (Table 1).

**TABLE 1:** Clinical demographic characteristics.

Characteristics	HD (*n* = 114)	CRC (*n* = 115)	BCD (*n* = 31)	NSCLC (*n* = 67)	BNC (*n* = 25)
Age, years	52.18 ± 8.39	56.48 ± 11.25	51.03 ± 13.73	54.01 ± 10.00	45.40 ± 10.47
Male, *n* (%)	46 (40.4%)	69 (60.0%)	19 (61.3%)	27 (40.3%)	10 (40.0%)
Smoking, *n* (%)					
Yes	32 (28.1%)	37 (32.2%)	8 (25.8%)	18 (26.9%)	7 (28.0%)
No	82 (71.9%)	78 (67.8%)	23 (74.2%)	49 (73.1%)	18 (72.0%)
Clinical stage, *n* (%)					
0	—	3 (2.6%)	—	—	—
I	—	10 (8.7%)	—	55 (82.1%)	—
II	—	20 (17.4%)	—	2 (3.0%)	—
III	—	50 (43.5%)	—	5 (7.5%)	—
IV	—	32 (27.8%)	—	5 (7.5%)	—
T stage, *n* (%)					
Tis	—	3 (2.6%)	—	—	—
T1	—	4 (3.5%)	—	44 (65.7%)	—
T2	—	12 (10.4%)	—	15 (22.4%)	—
T3	—	37 (32.2%)	—	6 (9.0%)	—
T4	—	59 (51.3%)	—	2 (3.0%)	—
Lymph nodes invasion, *n* (%)	—	82 (71.3%)	—	8 (11.9%)	—
Distant metastasis, *n* (%)	—	32 (27.8%)	—	5 (7.5%)	—
D50 value (IQR)	0.17 (0.08)	0.11 (0.10)	0.12 (0.11)	0.10 (0.10)	0.14 (0.13)
Shannon entropy (IQR)	8.68 (0.80)	8.07 (1.22)	8.31 (1.09)	8.20 (1.25)	8.61 (1.21)
Simpson index (IQR)	1.00 (0.01)	0.99 (0.01)	0.99 (0.01)	0.99 (0.01)	1.00 (0.00)

Abbreviations: BCD, benign colorectal disease; BNC, benign nodule controls; CRC, colorectal cancer; HD, healthy donors; IQR, interquartile range; NSCLC, nonsmall‐cell lung cancer.

### 3.2 Features of TCRβ Repertoires

**FIGURE 1:**
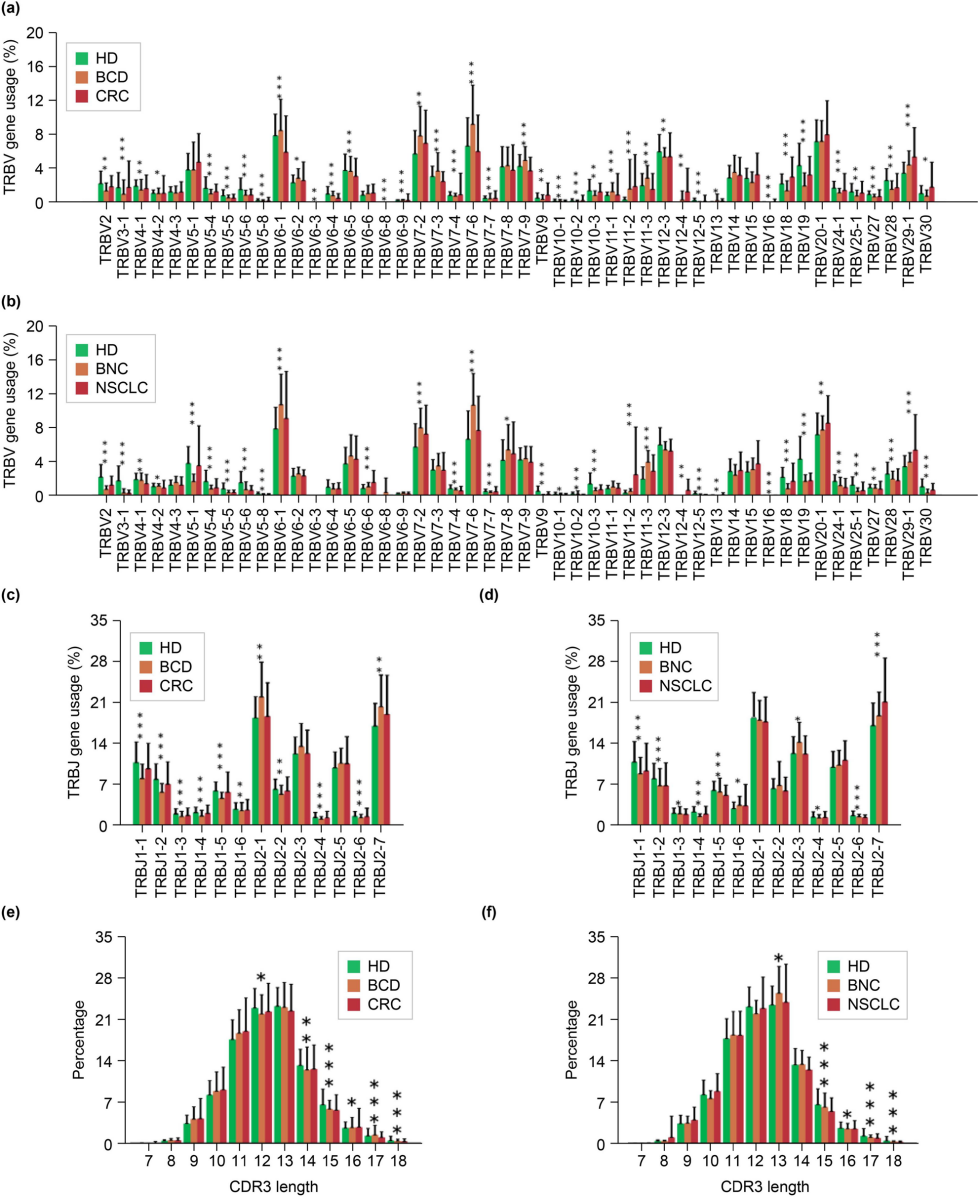
Vβ and Jβ gene usage and CDR3 amino acid sequence length distribution of the expressed TCRβ repertoires in the PB of HD and patients. (a) Comparison of Vβ genes in HD, BCD, and CRC patients; (b) comparison of Vβ genes in HD, BNC, and NSCLC patients; (c) comparison of Jβ genes in HD, BCD, and CRC patients; (d) comparison of Jβ genes in HD, BNC, and NSCLC patients; (e) comparison of the distribution of TCRβ CDR3 amino acid sequence lengths among HD, BCD, and CRC patients; (f) comparison of the distribution of TCRβ CDR3 amino acid sequence lengths among HD, BNC, and NSCLC patients. The asterisks indicate the *p* values of the Kruskal–Wallis test (*p < 0.05, **p < 0.01, ***p < 0.001). BCD, Benign colorectal disease; BNC, benign nodule controls; CRC, colorectal cancer; HD, healthy donors; NSCLC, nonsmall‐cell lung cancer.

Notable usage disparities were seen in 41 variable regions of the β chain (Vβ) and 11 joining regions of the β chain (Jβ) genes among the HD, BCD, and CRC groups (Figure 1a,c) and in 35 Vβ and 10 Jβ genes among the HD, BNC, and NSCLC groups (Figure 1b,d). A progressive increase in the use of the T‐cell receptor β variable (TRBV)11‐2, TRBV12‐4, and TRBV29‐1 genes in Vβ genes in the HD, CBD, and CRC groups, and of TRBV6‐6, TRBV11‐2, TRBV20‐1, and TRBV29‐1 in the HD, BNC, and NSCLC groups was noted. Conversely, a decreasing trend was observed in the use of TRBV5‐5, TRBV5‐8, TRBV6‐4, TRBV6‐5, and TRBV10‐1 genes in the HD, CBD, and CRC groups and of TRBV3‐1, TRBV4‐1, TRBV5‐6, TRBV5‐8, TRBV7‐4, TRBV10‐1, TRBV10‐2, TRBV12‐5, TRBV24‐1, TRBV27, and TRBV28 in the HD, BNC, and NSCLC groups. In these groups, the TCRβ CDR3 region's amino acid sequences had a similar length distribution, with a common peak at 13 amino acids (Figure 1e,f).

### 3.3 Patients With CRC and NSCLC Have Reduced PB‐TCR Diversity

**FIGURE 2:**
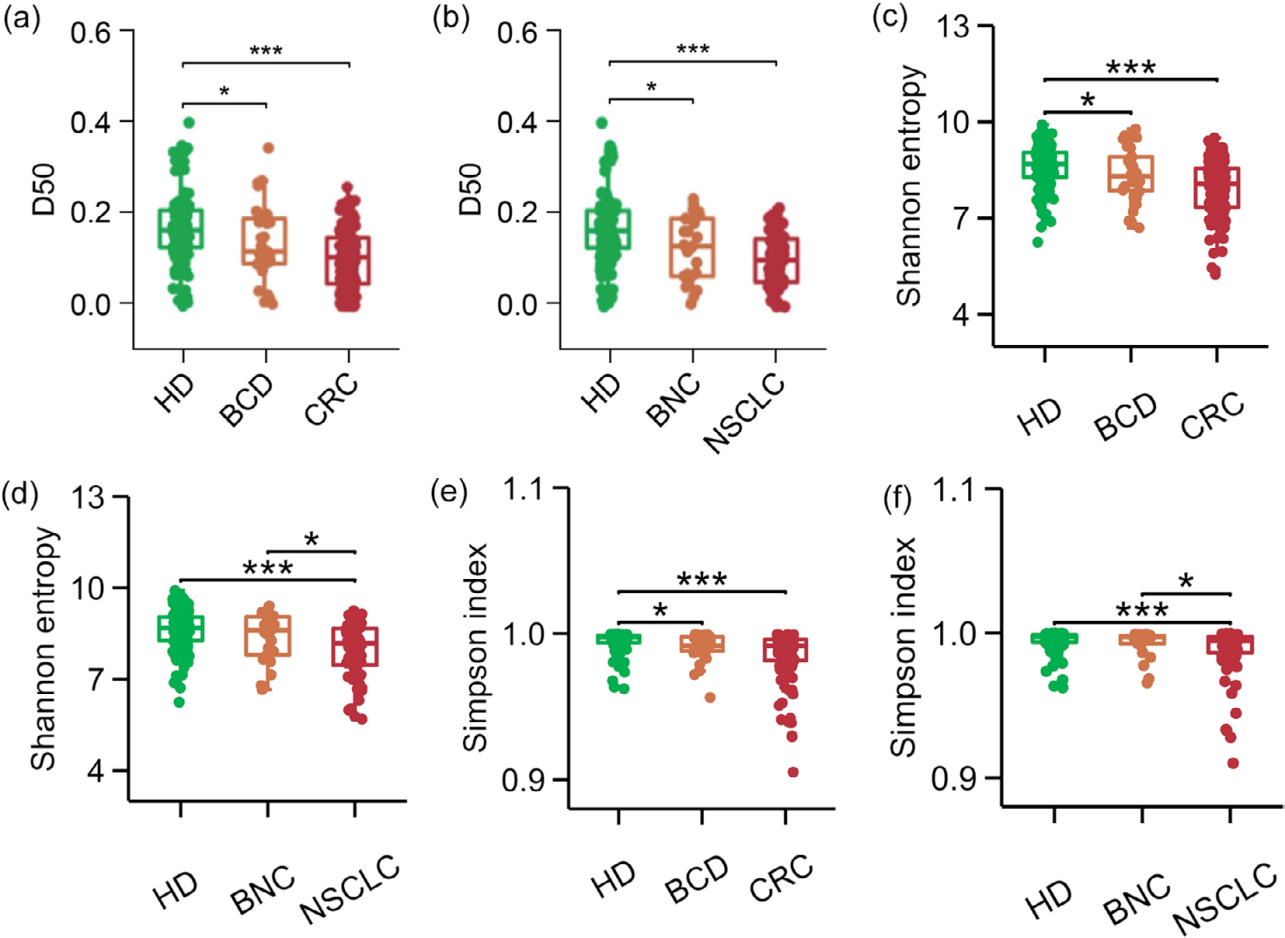
Comparison of PB TCR diversity among different groups. (a) Comparison of the D50 values among HD, BCD, and CRC patients. (b) Comparison of the D50 values among HD, BNC, and NSCLC patients. (c) Comparison of Shannon entropy among HD, BCD, and CRC patients. (d) Comparison of Shannon entropy among HD, BNC, and NSCLC patients. (e) Comparison of Simpson index among HD, BCD, and CRC patients. (f) Comparison of Simpson index among HD, BNC, and NSCLC patients. The asterisks indicate *p* values of the Wilcoxon test (*p < 0.05, ***p < 0.001). BCD, Benign colorectal disease; BNC, benign nodule controls; CRC, Colorectal cancer; HD, healthy donors; NSCLC, nonsmall‐cell lung cancer.

For the comparison between PB‐TCR diversity in HDs and patients, the results showed that both CRC and NSCLC patients showed similar significantly lower D50 values compared to HDs (both *p* < 0.001), whereas BCD patients and BNC patients exhibited modest decreases in TCR diversity compared to HDs (both *p* < 0.05) (Figure 2a,b). The variation trends of “Shannon entropy” and “Simpson index” among the HDs, BCD, and CRC groups are basically consistent with the D50 values (Figure 2c,e); the “Shannon entropy” and “Simpson index” of the HDs, BNC, and NSCLC groups showed that the TCR diversity of BNC was higher than that of NSCLC patients (both *p* < 0.05) (Figure 2d,f).

TCRβ CDR3 sequencing analysis indicated a significant hindrance in the number of TCRβ clone types in the CRC patients compared to the HDs (*p* < 0.05) and BCD patients (*p* < 0.05) (Figure S2a). NSCLC exhibited a lower number of TCRβ clone types compared to HDs (*p* < 0.001) (Figure S2b). The present study defined large TCRβ clones as unique TCRβ CDR3 clone types constituting > 1% of the overall sequences in an individual's repertoire [19]. Compared with the HDs, patients with CRC (*p* < 0.001) and NSCLC (*p* < 0.05) had a significantly higher number of big TCRβ clones (Figure S2c,d). We also found a statistically significant increase in the proportion of the largest TCRβ CDR3 clones in patients with CRC (*p* < 0.001) and NSCLC (*p* < 0.01) compared with HDs (Figure S2e,f). In addition, compared with HDs, a slight increase in the proportion of the largest TCRβ CDR3 clone was observed in patients with BCD (*p* < 0.05) (Figure S2e). It suggests that the progressive appearance of expanding TCRβ clones in the PB of individuals, ranging from healthy people to patients with benign diseases to patients with malignant tumors, is associated with a reduction in TCRβ diversity.

### 3.4 PB‐TCR Diversity of CRC and NSCLC Patients With Various Clinical and Pathological Features

The D50 value, Shannon entropy, and Simpson index were compared across patients with different sexes, age groups, clinical stages, T stages, distant metastases, and CEA levels. No significant differences (*p* > 0.05) were identified between patients with CRC and NSCLC. Nevertheless, a significant disparity in the D50 values, Shannon entropy, and Simpson index was noted between CRC patients aged < 60 years and those aged ≥ 60 years (*p* < 0.001, *p* < 0.001, and *p* = 0.008, respectively), while there were no significant differences in PB TCR diversity between different age groups in the patients with NSCLC (*p* > 0.05). Meanwhile, NSCLC patients with lymph node metastases had markedly lower D50 values, Shannon entropy, and Simpson index compared with those without lymph node metastasis (*p* < 0.01, *p* < 0.02, and *p* < 0.03, respectively), but this difference was not seen in CRC patients (*p* > 0.05). There was no significant difference in D50 values between smokers and nonsmokers of CRC patients as well as in NSCLC patients (both *p* > 0.05) (Table 2). CEA emerged as a valuable recurrence and metastasis marker for monitoring the progression of CRC and NSCLC. The results also revealed an association between the D50 values and CEA levels: a higher D50 and Shannon entropy level were found in patients with normal CEA in both CRC (*p* = 0.014 and 0.02, respectively) and NSCLC patients (*p* = 0.005 and 0.006, respectively), while the Simpson index did not show statistical significance (Table 2, Figure S3). The difference in D50 values between the two malignancies under different clinicopathological conditions was also compared. Table S1 shows that except for the D50 values between patients with positive lymph node metastasis (the D50 values of NSCLC patients were lower than that of CRC patients), there were no significant differences in other clinical characteristics.

**TABLE 2:** PB‐TCR diversity of CRC and NSCLC patients with different clinicopathological features.

Characteristics	CRC patients (*n* = 115)							NSCLC patients (*n* = 67)						
	*N*	D50 value	*p*	Shannon entropy	*p*	Simpson index	*p*	*N*	D50 value	*p*	Shannon entropy	*p*	Simpson index	*p*
Sex			0.42		0.58		0.84			0.60		0.40		0.27
Male	69	0.11 (0.10)		7.95 ± 0.88		0.99 (0.01)		27	0.10 ± 0.07		7.89 (1.570)		0.99 (0.02)	
Female	46	0.11 (0.11)		7.86 ± 0.95		0.99 (0.01)		40	0.10 ± 0.06		8.26 (1.14)		1.00 (0.01)	
Age (years)			< 0.001		< 0.001		0.008			0.06		0.15		0.37
< 60	69	0.13 (0.09)		8.15 ± 0.80		0.99 (0.01)		49	0.11 ± 0.06		8.28 (1.06)		0.99 (0.01)	
≥ 60	46	0.07 (0.10)		7.57 ± 0.95		0.99 (0.02)		18	0.08 ± 0.06		7.59 (1.74)		0.99 (0.02)	
Smoking			0.43		0.72		0.87			0.24		0.20		0.21
Yes	37	0.11 ± 0.07		7.99 (0.86)		0.99 (0.01)		18	0.09 ± 0.07		7.80 (1.70)		0.99 (0.02)	
No	78	0.10 ± 0.07		8.18 (1.24)		0.99 (0.02)		49	0.11 ± 0.06		8.25 (1.19)		0.99 (0.01)	
Clinical stage			0.40		0.35		0.33			0.24		0.49		0.49
0	3	0.10 ± 0.10		7.47 (0.00)		0.99 (0.00)		—	—		—		—	
I	10	0.12 ± 0.06		8.45 (0.86)		0.99 (0.01)		55	0.11 ± 0.06		8.26 (1.13)		1.00 (0.01)	
II	20	0.09 ± 0.07		7.69 (1.36)		0.99 (0.02)		2	0.08 ± 0.12		7.23 (0.00)		0.95 (0.00)	
III	50	0.12 ± 0.07		8.23 (1.35)		0.99 (0.02)		5	0.07 ± 0.05		7.56 (1.90)		0.99 (0.04)	
IV	32	0.09 ± 0.06		8.09 (1.29)		0.99 (0.01)		5	0.06 ± 0.06		7.19 (1.69)		0.98 (0.05)	
T stage			0.87		0.59		0.39			0.51		0.73		0.727
Tis	3	0.10 ± 0.10		7.47 (0.00)		0.99 (0.00)		—	—		—		—	
T1	4	0.10 ± 0.09		7.74 (2.02)		0.99 (0.03)		44	0.11 ± 0.06		8.29 (1.34)		1.00 (0.01)	
T2	12	0.13 ± 0.07		8.45 (0.64)		1.00 (0.01)		15	0.08 ± 0.04		8.05 (0.74)		0.99 (0.01)	
T3	37	0.11 ± 0.06		8.25 (0.91)		0.99 (0.02)		6	0.08 ± 0.07		7.94 (2.23)		0.99 (0.03)	
T4	59	0.10 ± 0.07		7.91 (1.27)		0.99 (0.01)		2	0.09 ± 0.03		8.19 (0.00)		1.00 (0.00)	
Lymph nodes			0.51		0.71		0.71			< 0.01		0.02		0.03
Positive	82	0.11 ± 0.07		8.12 (1.25)		0.99 (0.01)		8	0.05 ± 0.04		7.09 (1.01)		0.98 (0.05)	
Negative	33	0.10 ± 0.07		7.99 (1.22)		0.99 (0.02)		59	0.11 ± 0.06		8.28 (1.14)		1.00 (0.01)	
Distant metastasis			0.24		0.26		0.40			0.12		0.23		0.20
Yes	32	0.09 ± 0.06		8.09 (1.29)		0.99 (0.01)		5	0.06 ± 0.06		7.19 (1.69)		0.98 (0.05)	
No	83	0.11 ± 0.07		8.06 (1.26)		0.99 (0.02)		62	0.10 ± 0.06		8.26 (1.19)		1.00 (0.01)	
CEA			0.014		0.02		0.12			0.005		0.006		0.07
≤ 3.4 μg/L	47	0.12 ± 0.07		8.39 (1.41)		0.99 (0.01)		38	0.11 ± 0.06		8.29 (0.98)		1.00 (0.01)	
> 3.4 mg/L	68	0.09 ± 0.06		7.92 (1.39)		0.99 (0.02)		20	0.07 ± 0.05		7.49 (1.59)		0.99 (0.03)	

### 3.5 PB TCR Diversity as an Early Diagnostic Marker of CRC and NSCLC

**FIGURE 3:**
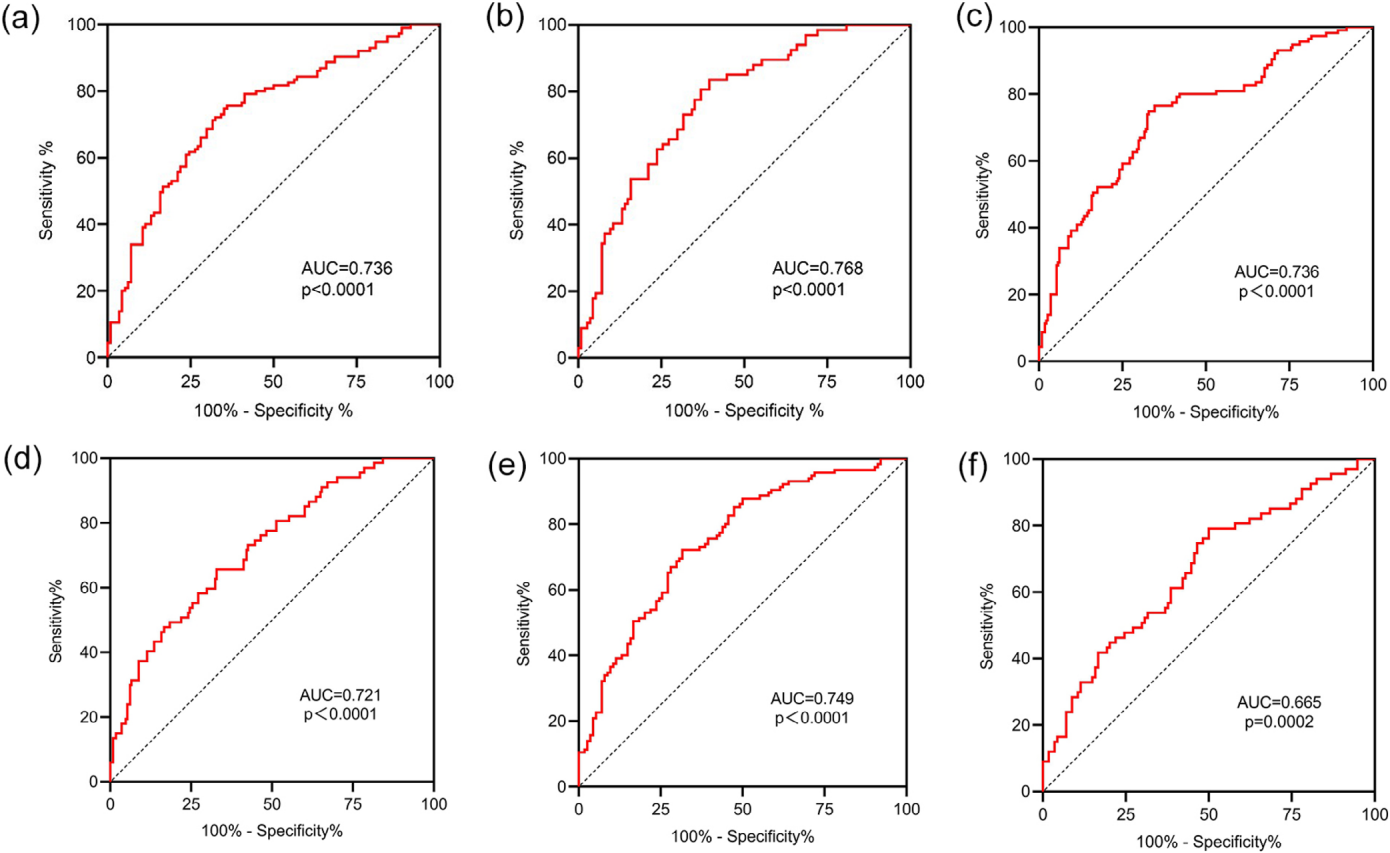
ROC curves of PB TCR diversity for CRC and NSCLC diagnosis. (a) ROC curve for the D50 value in CRC diagnosis, AUC = 0.736 (*p* < 0.0001), with a sensitivity of 71.30% and a specificity of 68.42%. (b) ROC curve analysis of the D50 value for NSCLC diagnosis, AUC = 0.768 (*p* < 0.0001), sensitivity was 83.58%, and specificity was 60.53%. (c) ROC curve for Shannon entropy in CRC diagnosis, AUC = 0.736 (*p* < 0.0001), with a sensitivity of 71.30% and specificity of 67.54%. (d) ROC curve analysis of Shannon entropy for NSCLC diagnosis, AUC = 0.721 (*p* < 0.0001), sensitivity was 65.67%, and specificity was 60.53%. (e) ROC curve for Simpson index in CRC diagnosis, AUC = 0.749 (*p* < 0.0001), with sensitivity of 71.30% and specificity of 68.42%. (f) ROC curve analysis of Simpson index for NSCLC diagnosis, AUC = 0.6652 (*p* = 0.0002), sensitivity was 61.19%, and specificity was 60.53%. ROC curve: Receiver operating characteristic curve; AUC: Area under the curve.

The area under the ROC curve (AUC) of D50 for CRC was 0.736 (95% CI 0.5941–0.7623, *p* < 0.0001), with a sensitivity of 71.30% and a specificity of 68.42% (Youden index = 0.3972) when the cutoff value was set at 0.145 (Figure 3a). The AUC for differentiating patients with NSCLC from BNC was 0.768 (95% CI 0.5135–0.6902, *p* < 0.0001), with a sensitivity of 83.58% and a specificity of 60.53% (Youden index = 0.4411) when the cutoff value was set at 0.156 (Figure 3b). The area under the ROC curve (AUC) of Shannon entropy for CRC was 0.736 (95% CI 0.6715–0.8001, *p* < 0.0001), with a sensitivity of 71.30% and a specificity of 67.54% (Youden index = 0.3884) at a cutoff value of 8.475 (Figure 3c). For differentiating NSCLC patients from BNC, the AUC was 0.721 (95% CI 0.6457–0.7966, *p* < 0.0001), with a sensitivity of 65.67% and specificity of 60.53% (Youden index = 0.2620) at a cutoff value of 8.575 (Figure 3d). The AUC of the Simpson index for CRC was 0.749 (95% CI 0.6861–0.8117, *p* < 0.0001), with a sensitivity of 71.30% and a specificity of 68.42% (Youden index = 0.3972) at a cutoff value of 0.9951 (Figure 3e). For differentiating NSCLC patients from BNC, the AUC was 0.6652 (95% CI 0.5831–0.7473, *p* = 0.0002), with a sensitivity of 61.19% and a specificity of 60.53% (Youden index = 0.2172) at a cutoff value of 0.9962 (Figure 3f).

### 3.6 PB TCR Diversity May Predict Neoadjuvant Therapy Efficacy

**FIGURE 4:**
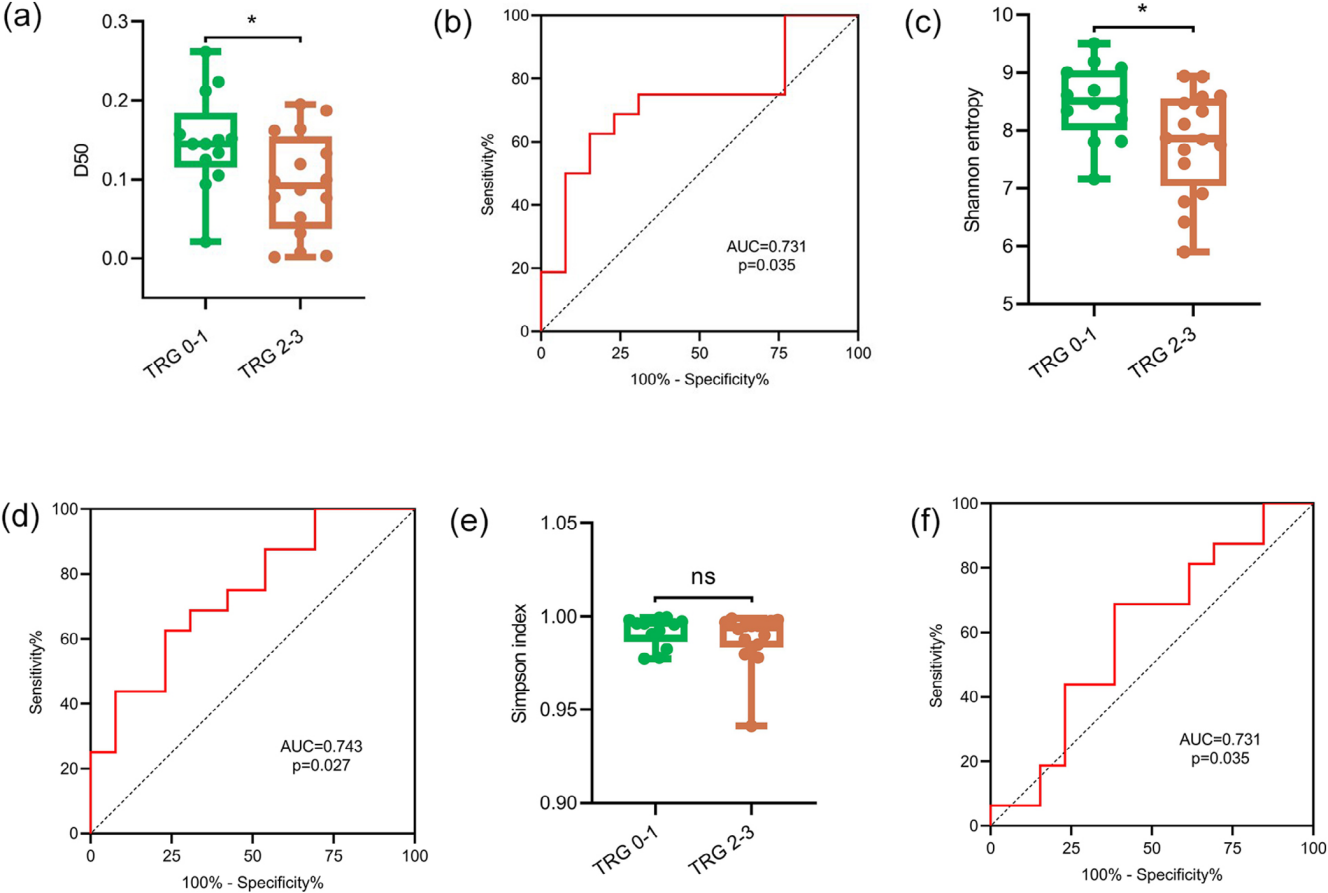
Analysis of PB TCR diversity as a potential marker for predicting the effectiveness of neoadjuvant therapy in patients with CRC. (a) Comparison of the D50 values in CRC patients with TRG grades 0–1 and 2–3. The asterisks indicate *p* values of the Wilcoxon test (*p < 0.05). (b) ROC curve of the D50 value for predicting the efficacy of neoadjuvant therapy. The AUC was 0.731 (*p =* 0.035), with a sensitivity of 68.75% and a specificity of 76.92%. (c) Comparison of Shannon entropy in CRC patients with TRG grades 0–1 and 2–3. The asterisks indicate the *p* values of the *t*‐test (*p < 0.05). (d) ROC curve of Shannon entropy for predicting the efficacy of neoadjuvant therapy. The AUC was 0.743 (*p* = 0.027), with a sensitivity of 62.50% and a specificity of 76.92%. (e) Comparison of Simpson index in CRC patients with TRG grades 0–1 and 2–3. The ns indicate *p* values of the Wilcoxon test (ns, no significance). (f) ROC curve of Simpson index for predicting the efficacy of neoadjuvant therapy. The AUC was 0.6010 (*p =* 0.357), with a sensitivity of 68.75% and a specificity of 61.54%. AUC, area under the curve; ROC curve, Receiver operating characteristic curve.

TRG is a widely accepted method for determining the efficacy of neoadjuvant therapy in patients with CRC (Table S2). Of 115 patients with CRC, 29 received neoadjuvant therapy (including three who received immunotherapy) and underwent postoperative TRG evaluation. Among these patients, 13 were classified as grades 0–1, whereas 16 were classified as grades 2–3 (Table S3). Types of pathology and organs involved were shown in Table S4. The study's results indicated a statistically significant difference in D50 values and Shannon entropy between individuals with TRG grades 0–1 and those with grades 2–3 (*p* = 0.027 and *p* = 0.025, respectively) (Figure 4a,c). However, no significant difference in the Simpson index was observed between the two groups of patients (*p* = 0.374) (Figure 4e). When the cutoff value was set to 0.122 (Youden index = 0.4567), the D50 value could effectively differentiate patients with TRG grading of 0–1 and 2–3 with the AUC of 0.731 (95% CI 0.4974–0.9182, *p* = 0.035), sensitivity of 68.75%, and specificity of 76.92% (Figure 4b). When the cutoff value was set to 8.155 (Youden index = 0.3942), the Shannon entropy could effectively differentiate patients with TRG grading of 0–1 and 2–3 with the AUC of 0.743 (95% CI 0.5629–0.9226, *p* = 0.027), a sensitivity of 62.50% and specificity of 76.92% (Figure 4d). When the cutoff value was set to 0.9954 (Youden index = 0.3029), the Simpson index could not effectively differentiate patients with TRG grading of 0–1 and 2–3 with the AUC of 0.6010 (95% CI 0.3854–0.8165, *p* = 0.357), sensitivity of 68.75%, and specificity of 61.54% (Figure 4f). The patients with NSCLC did not receive neoadjuvant therapy but underwent surgery directly after the tumor was found. Since TRG grading is an evaluation of the efficacy of neoadjuvant therapy, TRG grading was not evaluated for NSCLC.

## 4 Discussion

This study showed that various TRBV and T‐cell receptor β joining (TRBJ) genes were expressed differently among HDs and patients with CRC, NSCLC, or benign tumors. Moreover, the PB TCR diversity quantified by the D50 value was reduced in patients with CRC or NSCLC. When the D50 value was used for CRC and NSCLC diagnosis, the AUC achieved 0.736 and 0.768, respectively. Furthermore, the D50 value also exhibited its predictive value for the treatment efficacy of neoadjuvant therapy, with an AUC of 0.731.

Currently, commonly used TCR diversity detection methods include Shannon's entropy index, Simpson index, and the D50 value. The Shannon index is used to measure the diversity of TCR clones in a sample. This index can describe two aspects of information: species richness and evenness of individual distribution within a species. The higher the Shannon index, the higher the diversity of TCR [24, 25]. The Simpson index reflects the probability that two individuals are from the same class when randomly selected from the same sample. Therefore, the larger the value, the lower the diversity. It focuses more on reflecting the uniformity of clone species [23, 26]. The D50 is an indicator of clonality (clonal degree) that measures the uniformity of clonality. The higher the D50 value, the more evenly distributed the clones are, and the higher the diversity is. The lower the D50 value, the higher the clonality is, and the lower the diversity is, which means that some specific clones have been amplified [27, 28]. Therefore, D50 focuses on the diversity calculation of dominant unique clones. Previous studies did not systematically compare the advantages and disadvantages of these three indices. The present study used the D50 values, Shannon's entropy index, and Simpson index for TCR diversity analysis for the following reasons. Although the present study provided all three indicators, their trends of change were basically the same. Furthermore, the detection and calculation process of the D50 value can simultaneously analyze the amplification of TRBV clone fragments, which is conducive to the analysis and search for tumor‐specific TRBV fragments. In addition, the D50 value can be used as an indicator for evaluating human immunity, and a patent has been obtained for this purpose (patent number zl201910300069.9) [14]. Therefore, the discussion focuses more on the D50 value.

In this study, the length of the CDR3 amino acid sequence distributions was consistent across all groups during the TCRβ repertoire analysis in the PB. However, the use of the TRBV and TRBJ genes significantly differed between HDs and those with benign and malignant diseases. The use of TRBV11‐2 and TRBV29‐1 gradually increased during the onset and progression of CRC and NSCLC, whereas TRBV5‐8 and TRBV10‐1 gradually decreased. The results of the present study align with the study by Liu et al. [29], who also identified increased TRBV11‐2 in individuals with CRC compared with healthy controls. Further investigation of the shared TRBV genes in these two tumors could potentially lead to the identification of TCR sequences associated with both malignancies. These findings could be instrumental in advancing the development of T‐cell immunotherapies that specifically target these diseases.

The PB TCR diversity was further analyzed among HDs and patients with BCD, CRC, BNC, and NSCLC. The TCR diversity, as determined by the D50‐value, Shannon entropy, or Simpson index, was significantly lower in the PB of CRC patients aged over 60 years compared with those aged under 60 years, suggesting that the diversity of TCR in the PB of CRC patients decreases with age, which is in line with a previous study [14], where it was revealed that TCRβ diversity decreased with increasing age. The trend (yet not statistically significant) observed in the difference in PB TCR diversity between the two age groups among patients with NSCLC aligned with the trend observed in patients with CRC. The lack of statistical significance may be due to the small sample size. In addition, among the patients with NSCLC, individuals with lymph node metastasis exhibited lower TCR diversity than those without. However, given the limited number of patients with lymph node metastasis, further investigations are warranted. Smoking can affect TCR diversity, but the proportion of smokers was similar among all groups, including the NSCLC group. The low frequency could be attributed to the large proportion of women. Yet, most were in Xuanwei City, an area infamous for its high rate of lung cancer due to air pollution from coal mines and various industries [30]. This study compared the two malignancies and showed that, except for the D50 values between patients with positive lymph node metastasis (the D50 values of NSCLC patients were lower than that of CRC patients), there were no significant differences in other clinical characteristics. The possible reasons could be as follows: (1) when NSCLC patients have positive lymph node metastasis, they may be more prone to clonal expansion of T cells, resulting in a decrease in PB TCR diversity. This may be related to factors such as tumor antigen stimulation, chronic immune activation, and T‐cell exhaustion. (2) It may be due to the small number of NSCLC patients with positive lymph node metastasis (*n* = 8), leading to information bias. In future research, we will expand the sample size of this part to further determine whether this phenomenon exists.

This study also revealed a significant difference in the PB TCR diversity between cancer patients and HDs, consistent with the findings of previous studies [14, 29, 31]. The PB TCR diversity was significantly reduced in BCD and BNC patients compared to HDs. Moreover, the Shannon entropy and Simpson index of BNC were lower than those of NSCLC, suggesting a gradual decline in TCR diversity from HDs to patients with benign disease and then to patients with malignant tumors. It also indicates that the three TCR diversity indicators can complement each other to some extent. Moreover, reduced TCR diversity, reduced number of TCR clones, and expansion of large TCRβ clones may result from the antigen‐driven T‐cell clonal expansion. Thus, the observations in this study imply that differences in TCR diversity among groups may be attributed to the substantial expansion of certain CDR3 sequences and the concomitant reduction in other sequences in patients with CRC and NSCLC. It explains the aforementioned changes in the TRBV and TRBJ genes. Zhou et al. demonstrated that persistent tumor antigens could alter antigen‐specific T cells [32]. The expansion of large TCRβ clones may be attributed to prolonged exposure to tumor antigens, which can lead to personalized immune responses in individuals. The results indicated that TCR diversity could be a biomarker for distinguishing patients with CRC, NSCLC, and HDs.

Diagnosing solid tumors such as CRC and NSCLC involves invasive procedures such as colonoscopy and biopsy. These methods can cause discomfort and pain in patients undergoing screening, and there is a need to develop noninvasive methods for early diagnosis. An ROC curve was constructed to investigate the TCR diversity in PB as an early diagnostic tool for CRC and NSCLC. Comparing the ROC curves analyzed by the D50 value, Shannon entropy, and Simpson index, in the diagnosis of CRC, under the condition that the sensitivity of the three indicators is the same, the specificity of Shannon entropy is lower than that of the D50 value, while the specificity of the Simpson index is the same as that of the D50 value. In the diagnosis of NSCLC, under the condition that the specificity of the three indicators is the same, the sensitivity of both Shannon entropy and Simpson index is not as good as that of the D50 value. Therefore, we believe that the D50 value has more value in assisting diagnosis compared with other indicators. Still, while D50 shows promise in distinguishing cancer patients from healthy individuals, its AUC values indicate moderate diagnostic performance. Therefore, D50 should be used in combination with other biomarkers or clinical parameters for optimal diagnostic accuracy. TCRβ repertoire with high diversity is characterized by a D50 value greater than 0.10 [33]. These findings indicated that setting a D50 cutoff point of 0.145 could be a dependable approach for distinguishing between CRC and HD. Similarly, a cutoff of 0.156 could reliably distinguish NSCLC from HD. It suggests that the two models have a moderate ability to identify cancer patients accurately. An AUC of 1.0 indicates perfect discrimination, whereas an AUC of 0.5 indicates that the test has no better discrimination than pure chance. An AUC > 0.8 is usually considered of clinical significance, whereas those < 0.8 are considered of limited clinical utility. The AUCs in the present study were 0.736–0.768 for identifying cancer from HDs. These results suggest that using the D50 value alone could be of limited value [34]. Nevertheless, it suggests that the D50 value could be combined in a model alongside other clinical indexes to achieve optimal discrimination for cancer patients. Future studies will examine such models. Nevertheless, a gradual increase was observed in TRBV11‐2, TRBV12‐4, and TRBV29‐1 genes in the Vβ gene in the HD, CBD, and CRC groups, and a gradual increase was observed in TRBV6‐6, TRBV11‐2, TRBV20‐1, and TRBV29‐1 in the HD, BNC, and NSCLC groups. Hence, the D50 value and the changes in TRBV11‐2, TRBV12‐4, TRBV29‐1, TRBV6‐6, TRBV11‐2, TRBV20‐1, and TRBV29‐1 could be used together to diagnose CRC and NSCLC. Large‐scale validation studies will be necessary to determine whether these fragments are tumor‐specific fragments that can be used for diagnosis.

Several studies have identified biological markers in PB with early diagnostic potential for tumors. These markers include cell‐free DNA (cfDNA), circulating tumor cells (CTCs), circulating tumor DNA (ctDNA), and circulating microRNAs (miRNAs). With the rapid development of single‐cell and high‐throughput sequencing technologies, cfDNA or CTCs in liquid biopsies are widely used [35, 36, 37]. Although they show promising potential for cancer diagnosis, recent research has indicated that the majority of mutations detected in plasma cfDNA originate from leukocytes rather than cancer cells [38]; thus, the specificity of cfDNA‐based approaches is questionable. The low concentration of CTCs in the blood [39] and their short half‐life, which typically ranges from 1 to 2 h, limit their clinical use [40]. The proportion of ctDNA in the blood of cancer patients is typically below 0.1% [41], which presents challenges for detection because the process is complex and costly. Furthermore, ctDNA‐based tests' sensitivity and specificity to accurately identify tumors in their early stages are limited [41, 42]. The outcomes of circulating miRNA have significant potential as a novel biomarker for the early detection of CRC. However, quantitatively detecting miRNAs is challenging because of their inherent properties [43]. In the present study, the D50 value was used for the early detection of CRC and NSCLC via next‐generation sequencing (NGS) of the TCR sequence. This circumvents the limitations of insufficient levels of the aforementioned indicators in the blood. Furthermore, the detection method is relatively well established and straightforward. Although the diagnostic specificity and sensitivity of the D50 may not be as high as some other early diagnostic indicators, it is easier to detect. Research has shown that combining multiple indicators can enhance the specificity and sensitivity of early tumor diagnosis [44]. Since the BCD and BNC patients and HDs in this study did not undergo CEA testing, a combined diagnostic value study of D50 and CEA was not conducted. In future research, integrating the D50 with other blood markers may enhance the specificity and sensitivity of early diagnosis of these diseases.

Neoadjuvant therapy, which includes the use of immunotherapy, is administered before surgical intervention [45]. The efficacy of this treatment approach can be evaluated through RECIST version 1.1 or postoperative assessment of TRG. Consequently, there is currently a lack of objective and noninvasive evaluation approaches to accurately measure treatment efficacy preoperatively. Mismatch repair deficiency and tumor mutational burden (TMB) are potential predictors of immunotherapy efficacy [46, 47]. However, the identification of these biomarkers requires the acquisition of tumor tissue. Because of the utilization of various bioinformatics algorithms in current whole‐exome sequencing techniques, NGS panels present a formidable task in determining a universally applicable threshold for TMB that can effectively guide the administration of PD‐1 inhibitors in patients with metastatic CRC (mCRC) [45]. A novel approach for assessing the efficacy of neoadjuvant therapy is required to find patients for whom neoadjuvant treatment may be beneficial before surgery. Certain DNA methylation and miRNAs found in tumors may function as predictive markers for the efficacy of neoadjuvant therapy [48, 49, 50, 51]. Nevertheless, all predictors were obtained from the tumor tissue. Caramés et al. [51] employed miR‐31 as a prognostic indicator to assess the effectiveness of neoadjuvant therapy in patients diagnosed with locally advanced rectal cancer. In their study, the area under the ROC curve was 0.71 (*p* = 0.001), the specificity of the predictor was 76.3%, whereas the sensitivity was relatively low at 60.8%. Previous studies, except that by Caramés et al. [51], have not provided clear indications of the sensitivity and specificity of predicting the curative effects. Neoadjuvant chemoradiotherapy has been reported to be more effective in rectal cancer patients with high CD8+ TIL TCR diversity, both before and after treatment, as determined by the TRG [52]. In addition, the use of biopsy tissue from patients with CRC for immune‐score analysis is a valuable predictor of chemotherapy sensitivity and survival time [53]. It highlights the crucial function of the immune system in determining the efficacy of chemotherapy treatment in patients. However, all the methods require a pathological biopsy, which causes discomfort and inconvenience to patients. Therefore, a comprehensive analysis was conducted to determine the potential of TCR diversity in the PB as a prognostic indicator for the efficacy of neoadjuvant therapy. The study suggested that patients with lower TCR diversity in their PB before treatment were likelier to have elevated TRG scores. The ROC curve was generated to evaluate the prognostic capacity of the D50 value for neoadjuvant therapy (AUC = 0.731, *p* = 0.035), and the model had 68.75% sensitivity and 76.92% specificity. Although Shannon entropy also has the potential to evaluate the prognosis of neoadjuvant therapy (AUC = 0.743, *p* = 0.027), its sensitivity is lower than that of the D50 value. Meanwhile, the Simpson index is unable to evaluate the prognosis of neoadjuvant therapy (AUC = 0.6010, *p* = 0.357). Therefore, the D50 value is the best choice among the three indicators for evaluating the prognosis of neoadjuvant therapy. These findings indicate that the efficacy of neoadjuvant therapy may be better in patients with high TCR diversity in the PB. Furthermore, the reported specificity and sensitivity surpassed that of Caramés et al. The proposed method obviates the need to acquire tumor tissues. In terms of treatment decision‐making, the results showed that when the cutoff point is set to 0.122, the D50 value can effectively distinguish patients with TRG grades 0–1 and 2–3. Therefore, we could stratify and screen the population that can benefit from neoadjuvant therapy by understanding whether the D50 value of patients is higher than 0.122 before neoadjuvant therapy to avoid ineffective treatment for some patients who cannot benefit and reduce the pain caused by chemotherapy.

The immune system helps inhibit tumor proliferation and metastasis [15]. Changes and differences in TCR diversity, such as variation in the frequency of V and J gene use, distribution of CDR3 length, and differences in amino acid use in CDR3, are associated with the cancer patient's prognosis [54, 55, 56]. However, their ability to predict the effectiveness of chemotherapy and targeted therapy is debatable [57, 58], and small sample sizes limit these investigations. In the present study, greater sample sizes were included, and analysis indicated that patients with normal CEA levels demonstrated a notably higher D50 value and Shannon entropy compared with those with increased CEA levels. Although the Simpson index showed no significance among CRC patients, the same trend as that of the D50 and Shannon entropy could be observed. The lack of statistical significance may be related to the low sensitivity of this indicator. Previous investigations demonstrated a significant correlation between elevated CEA levels and the probability of an unfavorable prognosis in patients with CRC and NSCLC [19, 20, 21]. Here, a reduction in PB TCR diversity was found in patients with higher CEA levels prior to initiating treatment, indicating that PB TCR diversity may indicate tumor recurrence and metastasis and is a prognostic factor for CRC and NSCLC patients. However, additional validation via prolonged follow‐up observations is imperative to ensure the accuracy and reliability of these findings.

Nevertheless, the D50 had a higher sensitivity for NSCLC but lower specificity compared with CRC. Although the literature can provide some insights, the present study was not designed to answer that question. Nevertheless, it can be hypothesis‐generating. First, CRC and NSCLC have different tumor microenvironments and immune evasion mechanisms. CRC tends to present fewer immune checkpoint molecules compared to NSCLC, whereas NSCLC tends to have a more immunosuppressive tumor microenvironment due to the frequent upregulation of immune checkpoint molecules, such as PD‐L1 [59]. This immune evasion mechanism allows the tumor to suppress a broad immune response, leading to a scenario where only a few dominant T‐cell clones expand in response to the tumor. These dominant clonotypes are more easily detected by the D50 analysis, enhancing the sensitivity of the test in NSCLC. Nevertheless, this clonal expansion of a limited number of T cells also means that the immune response is not as diverse, leading to immune escape in some cases and allowing false positives to emerge from nonspecific immune activity. It could explain the lower specificity in NSCLC, as the immune system may be less targeted and allow other factors (e.g., inflammation, nontumor‐related immune activation) to be picked up by the test. In a previous study, the analysis of T‐cell clones between normal lung tissue and NSCLC revealed a high degree of similarity [60], which may be a result of persistent exposure to pathogens rather than a response to specific tumor antigens, further supporting this hypothesis. CRC, on the other hand, often exhibits a more stable and localized immune response, particularly due to the presence of gut‐associated lymphoid tissue (GALT) [61]. The immune system in the gut is constantly balancing tolerance with surveillance, which can lead to a more polyclonal and diverse immune response. This broader immune activity may result in lower sensitivity because the D50 value, reflecting the diversity of TCR clones, is less dominated by a few tumor‐specific clones. CRC, particularly in the context of the gut immune system, involves a more delicate balance between tolerance to commensal microbiota and surveillance against malignant cells. This tolerance mechanism can contribute to a more controlled immune response, reducing false positives and increasing specificity. However, this balance may also limit the strength of the response, leading to lower sensitivity in detecting early‐stage or less immunogenic tumors. A second factor could be the TMB and antigen presentation. NSCLC typically has a higher TMB than CRC [62]. The larger number of mutations leads to the presentation of more neoantigens, which can trigger strong clonal expansion of a few dominant T‐cell populations [63]. This high level of clonal expansion contributes to the higher sensitivity seen in NSCLC, as these few clonotypes are efficiently detected. In contrast, CRC generally has a lower TMB, except in a few cases, like those with microsatellite instability (MSI). As a result, the TCR response is more diverse and distributed, which could dilute the immune response detected by the D50 value, leading to lower sensitivity but more targeted and specific detection of tumor antigens.

This study still had some limitations. First, the number of patients with BCD and BNC included in this study was relatively small due to low awareness of regular check‐ups in the study area, resulting in delayed detection of such diseases. Second, the diagnostic performance of D50 for CRC and NSCLC patients only showed moderate diagnostic capability with an AUC greater than 0.7 in the internal data set; the effectiveness still requires further validation in larger external cohorts. Third, a combination of multiple biomarkers could be beneficial for improving efficiency. However, the biomarkers for BCD patients, BNC patients, and HDs were not evaluated in this study. Fourth, only three of the participants were treated with ICIs, and the sample size was small. Therefore, the efficacy assessment of immunotherapy could not be clarified. Finally, since NSCLC patients underwent direct surgery without neoadjuvant therapy, the efficacy of neoadjuvant therapy in NSCLC patients could not be analyzed. The follow‐up was short, and most patients did not experience progression events. Therefore, a prognostic model could not be built. Future studies should consider integrating D50 with Shannon entropy, Simpson index, and clinical parameters such as TRB gene usage and CEA levels to develop a comprehensive predictive model for cancer classification and treatment assessment.

In conclusion, PB TCR D50 values may be used as a quantitative measure for patients with CRC and NSCLC. Moreover, the D50 value is superior to Shannon entropy and Simpson index in aspects such as tumor diagnosis and prognosis prediction of neoadjuvant therapy. The significant differences in PB TCR diversity observed in cancer patients and HDs indicated its great potential in tumor diagnosis, treatment evaluation, and prognostic prediction. The results provide insights for developing effective tools to assess the efficacy of immunotherapy, fostering future research in this field.

## Author Contributions

**Jilong Ma:** data curation (equal), writing – original draft (equal), writing – review and editing (equal). **Yuanbiao Wang:** data curation (equal), writing – original draft (equal), writing – review and editing (equal). **Zhixin Zhang:** formal analysis (equal), writing – original draft (equal), writing – review and editing (equal). **Xinyi Cai:** formal analysis (equal), writing – original draft (equal), writing – review and editing (equal). **Xudong Xiang:** formal analysis (equal), writing – original draft (equal), writing – review and editing (equal). **Yan Chen:** methodology (equal), writing – original draft (equal), writing – review and editing (equal). **Fengqiong Sun:** methodology (equal), writing – original draft (equal), writing – review and editing (equal). **Jian Dong:** methodology (equal), writing – original draft (equal), writing – review and editing (equal).

## Ethics Statement

This work has been carried out in accordance with the Declaration of Helsinki (2000) of the World Medical Association. This study was approved by the Medical Ethics Committee of Yunnan Cancer Hospital (Grant No. KYLX2022201).

## Consent

Prior to sample collection, all participants completed an informed consent form.

## Conflicts of Interest

The authors declare that they have no competing interests. A patent related to the use of the D50 value as an indicator for evaluating human immunity has been obtained (patent number ZL201910300069.9).

## Supporting information


**Figure S1.** Patient flowchart.

**Figure S2.** TCRβ CDR3 sequencing analysis. (a) Comparison of the number of TCRβ clone types in HD, BCD, and CRC patients. (b) Comparison of the number of TCRβ clone types in HD, BNC, and NSCLC patients. (c) Comparison of the number of big TCRβ clones larger than 1% in HD, BCD, and CRC patients. (d) Comparison of the number of big TCRβ clones larger than 1% in HD, BNC, and NSCLC patients. (e) Comparison ratio of the largest clones of TCRβ CDR3 in HD, BCD, and CRC patients. (f) Comparison ratio of the largest clones of TCRβ CDR3 in HD, BNC, and NSCLC patients. The asterisks indicate *p* values of the Wilcoxon test (*p < 0.05, **p < 0.01, ***p < 0.001). BCD, benign colorectal disease; BNC, benign nodule controls; CRC, colorectal cancer; HD, healthy donors; NSCLC, nonsmall‐cell lung cancer.

**Figure S3.** The correlation between CEA and the PB TCR diversity in CRC and NSCLC patients. (a) Comparison of D50 values between CRC patients with normal CEA levels and those with elevated levels. (b) Comparison of D50 values between NSCLC patients with normal CEA levels and those with elevated levels. (c) Comparison of Shannon entropy between CRC patients with normal CEA levels and those with elevated levels. (d) Comparison of Shannon entropy between NSCLC patients with normal CEA levels and those with elevated levels. (e) Comparison of Simpson index between CRC patients with normal CEA levels and those with elevated levels. (f) Comparison of Simpson index between NSCLC patients with normal CEA levels and those with elevated levels. The asterisks and ns indicate *p* values of the Wilcoxon test (*p < 0.05, **p < 0.01, ns, no significance). CEA, carcinoembryonic antigens; CRC, colorectal cancer; NSCLC, nonsmall‐cell lung cancer.

**Table S1.** Comparison of PB TCR diversity in different clinical characteristics between CRC and NSCLC patients.
**Table S2.** The tumor regression grade (TRG) (modification of Ryan et al.).
**Table S3.** Characteristics of patients who received neoadjuvant therapy.
**Table S4.** Types of pathology and organs involved.

Data S1.

## Data Availability

The data supporting the conclusions of this research may be obtained upon request to the corresponding author. The data are inaccessible to the public owing to privacy or ethical constraints.
