# Supplementary figures and images for: Peripheral Blood T‐Cell Receptor Repertoire Diversity as a Potential Biomarker in the Diagnosis and Treatment Evaluation of Colorectal and Lung Cancers: A Prospective Observational Study
Source: Cancer Med. 2025 May 19;14(10):e70937. doi: 10.1002/cam4.70937 (PMC12086972; doi:10.1002/cam4.70937)

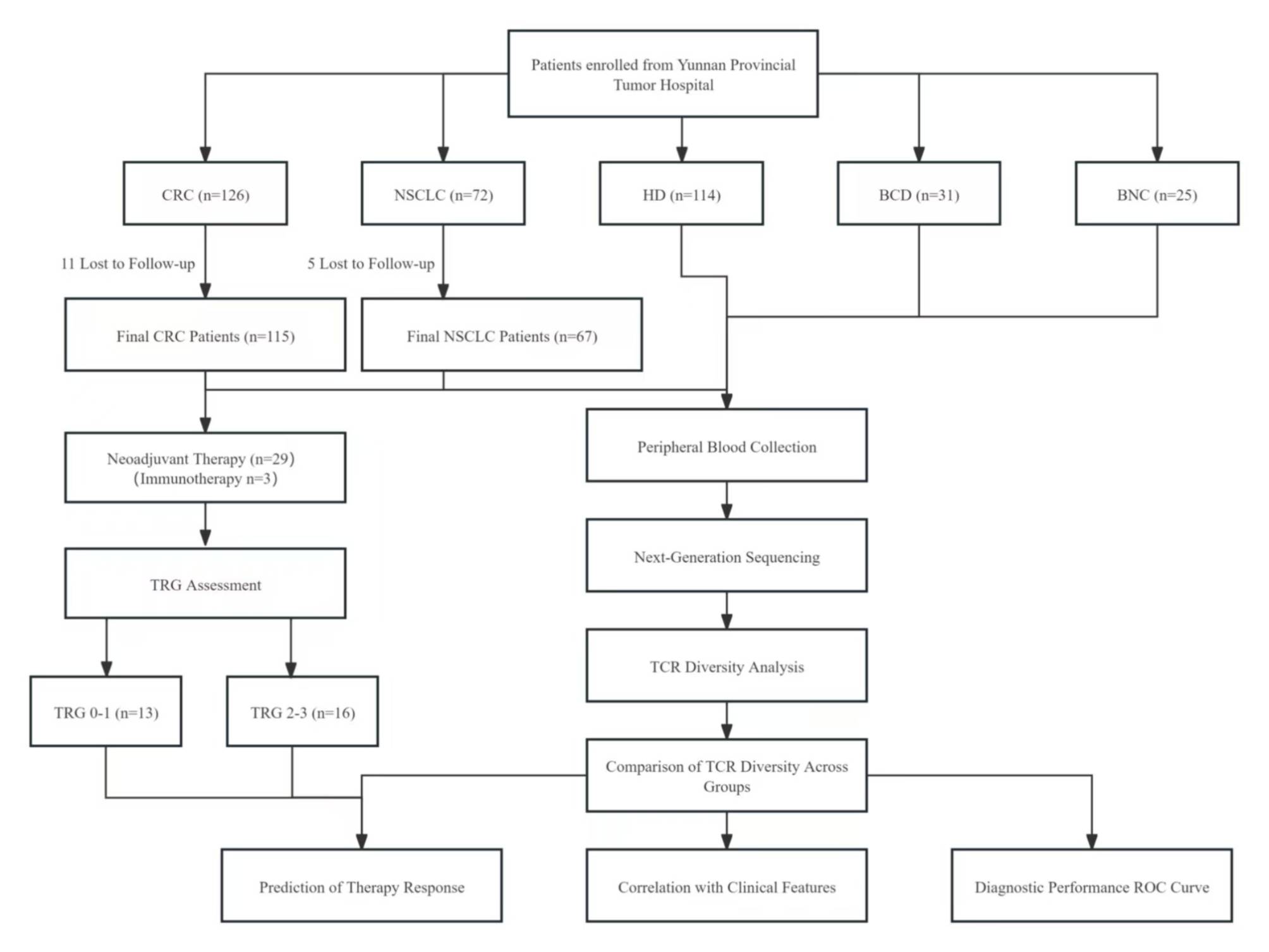

Supplement: Supplementary file 1 — Figure S1. Patient flowchart. [file CAM4-14-e70937-s001.jpg]

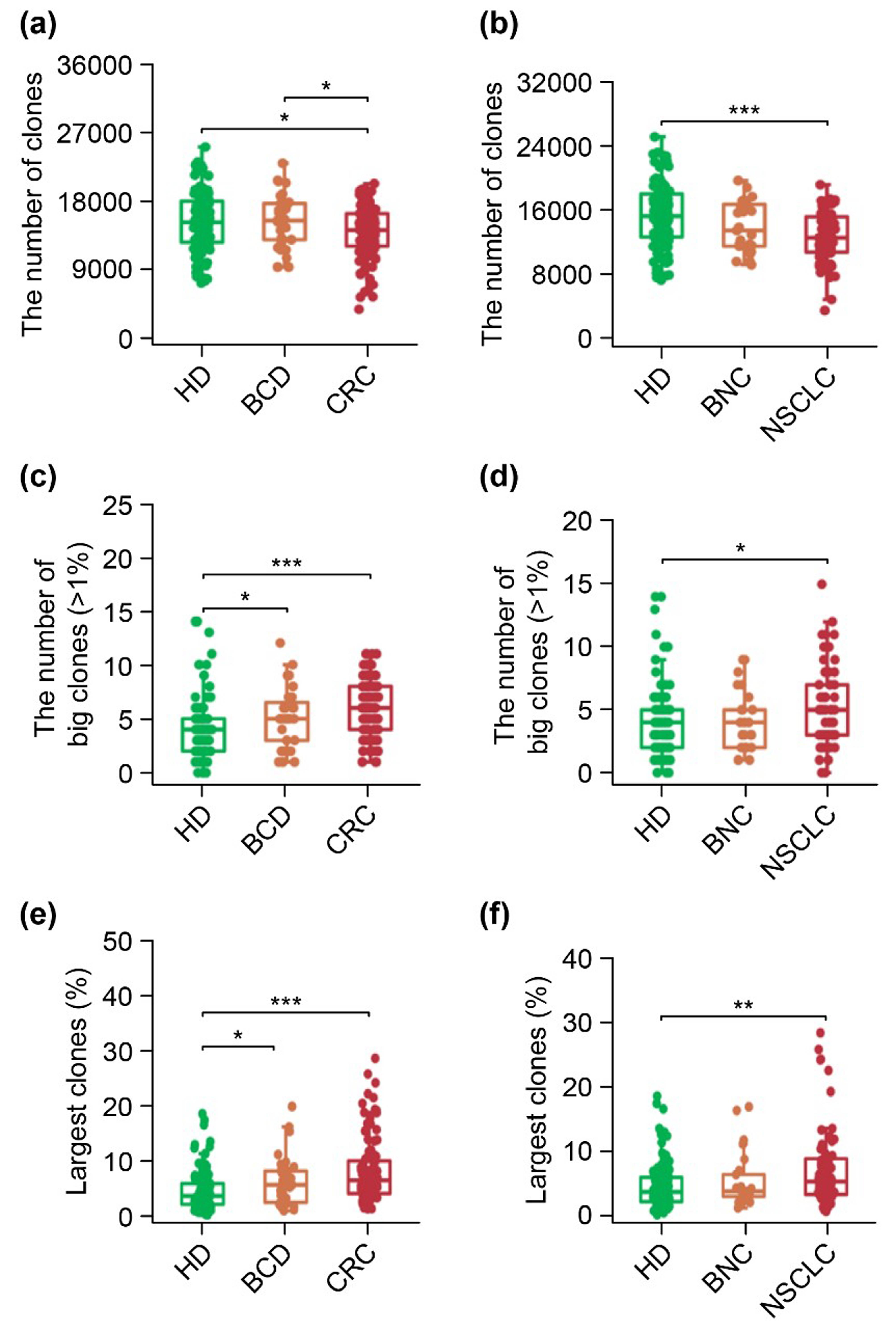

Supplement: Supplementary file 2 — Figure S2. TCRβ CDR3 sequencing analysis. (a) Comparison of the number of TCRβ clone types in HD, BCD, and CRC patients. (b) Comparison of the number of TCRβ clone types in HD, BNC, and NSCLC patients. (c) Comparison of the number of big TCRβ clones larger than 1% in HD, BCD, and CRC patients. (d) Comparison of the number of big TCRβ clones larger than 1% in HD, BNC, and NSCLC patients. (e) Comparison ratio of the largest clones of TCRβ CDR3 in HD, BCD, and CRC patients. (f) Comparison ratio of the largest clones of TCRβ CDR3 in HD, BNC, and NSCLC patients. The asterisks indicate p values of the Wilcoxon test (*p < 0.05, **p < 0.01, ***p < 0.001). BCD, benign colorectal disease; BNC, benign nodule controls; CRC, colorectal cancer; HD, healthy donors; NSCLC, nonsmall‐cell lung cancer. [file CAM4-14-e70937-s005.jpg]

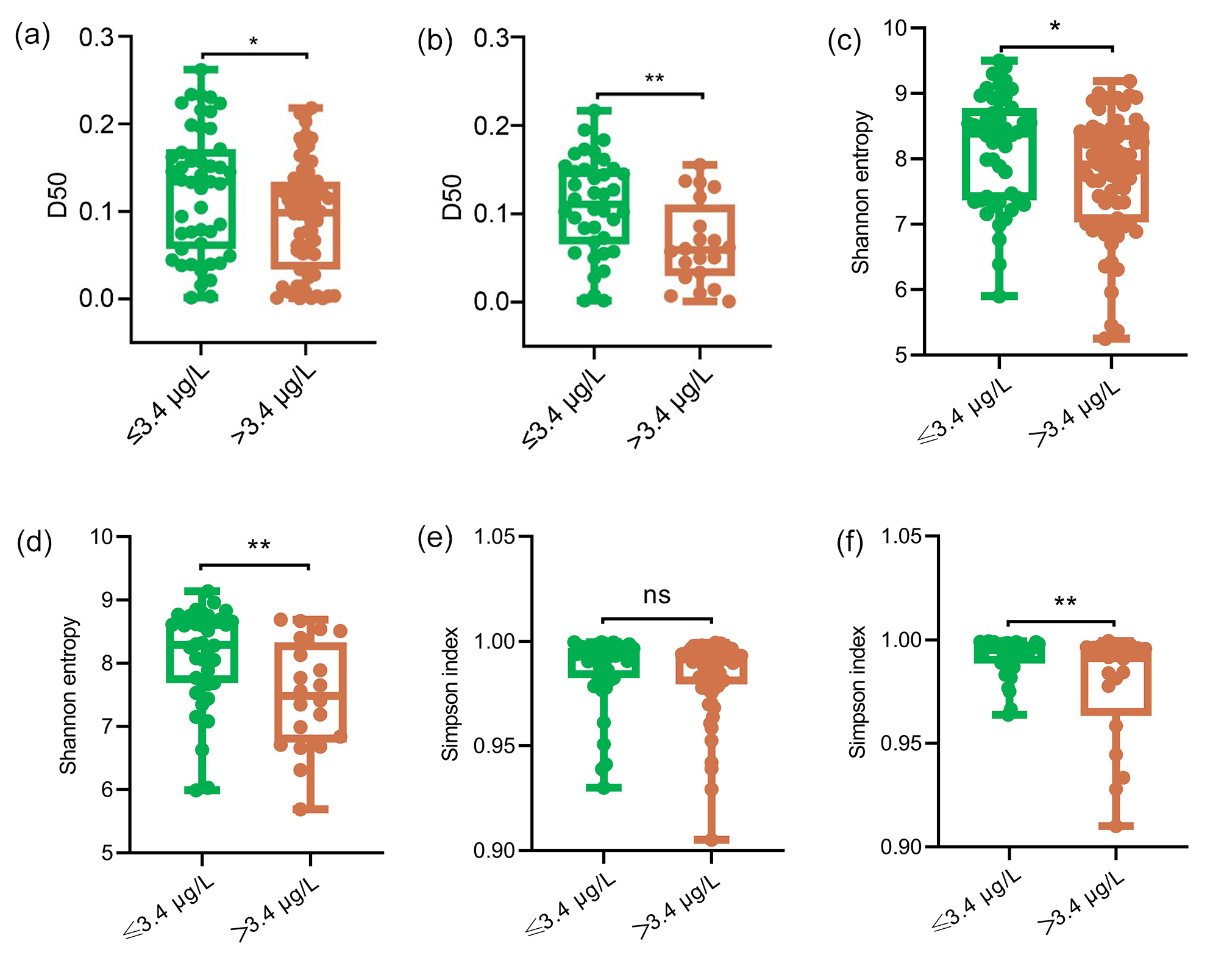

Supplement: Supplementary file 3 — Figure S3. The correlation between CEA and the PB TCR diversity in CRC and NSCLC patients. (a) Comparison of D50 values between CRC patients with normal CEA levels and those with elevated levels. (b) Comparison of D50 values between NSCLC patients with normal CEA levels and those with elevated levels. (c) Comparison of Shannon entropy between CRC patients with normal CEA levels and those with elevated levels. (d) Comparison of Shannon entropy between NSCLC patients with normal CEA levels and those with elevated levels. (e) Comparison of Simpson index between CRC patients with normal CEA levels and those with elevated levels. (f) Comparison of Simpson index between NSCLC patients with normal CEA levels and those with elevated levels. The asterisks and ns indicate p values of the Wilcoxon test (*p < 0.05, **p < 0.01, ns, no significance). CEA, carcinoembryonic antigens; CRC, colorectal cancer; NSCLC, nonsmall‐cell lung cancer. [file CAM4-14-e70937-s004.jpg]
