# Supplementary material for: Peripheral Blood T‐Cell Receptor Repertoire Diversity as a Potential Biomarker in the Diagnosis and Treatment Evaluation of Colorectal and Lung Cancers: A Prospective Observational Study
Source: Cancer Med. 2025 May 19;14(10):e70937. doi: 10.1002/cam4.70937 (PMC12086972; doi:10.1002/cam4.70937)
Supplement: Supplementary file 4 — Table S1. Comparison of PB TCR diversity in different clinical characteristics between CRC and NSCLC patients. Table S2. The tumor regression grade (TRG) (modification of Ryan et al.). Table S3. Characteristics of patients who received neoadjuvant therapy. Table S4. Types of pathology and organs involved. [file CAM4-14-e70937-s003.docx]

**Table S1. Comparison of PB TCR Diversity in Different Clinical Characteristics Between CRC and NSCLC Patients**

| Characteristics | CRC patients (N = 115) | | NSCLC patients (N = 67) | | p-value |
| --- | --- | --- | --- | --- | --- |
|  | **N** | **D50 Value** | **N** | **D50 value** |  |
| Sex |  |  |  |  |  |
| Male | 69 | 0.11±0.07 | 27 | 0.10±0.07 | 0.35 |
| Female | 46 | 0.11(0.11) | 40 | 0.10(0.10) | 0.66 |
| Age (years) |  |  |  |  |  |
| <60 | 69 | 0.12±0.07 | 49 | 0.11±0.06 | 0.19 |
| ≥60 | 46 | 0.07(0.10) | 18 | 0.06(0.11) | 0.87 |
| SMOKING |  |  |  |  |  |
| YES | 37 | 0.11±0.07 | 18 | 0.09±0.07 | 0.17 |
| NO | 78 | 0.10±0.07 | 49 | 0.11±0.06 | 0.77 |
| Clinical stage |  |  |  |  |  |
| 0 | 3 | 0.10±0.10 | - | - | - |
| I | 10 | 0.12±0.06 | 55 | 0.11±0.06 | 0.61 |
| II | 20 | 0.09±0.07 | 2 | 0.08±0.12 | 0.94 |
| III | 50 | 0.12±0.07 | 5 | 0.07±0.05 | 0.16 |
| IV | 32 | 0.09±0.06 | 5 | 0.06±0.06 | 0.28 |
| T-stage |  |  |  |  |  |
| Tis | 3 | 0.10±0.10 | - | - | - |
| T1 | 4 | 0.10±0.09 | 44 | 0.11±0.06 | 0.75 |
| T2 | 12 | 0.13±0.07 | 15 | 0.08±0.04 | 0.59 |
| T3 | 37 | 0.11±0.06 | 6 | 0.08±0.07 | 0.45 |
| T4 | 59 | 0.10±0.07 | 2 | 0.09±0.03 | 0.89 |
| Lymph nodes |  |  |  |  |  |
| Positive | 82 | 0.11±0.07 | 8 | 0.05±0.04 | 0.02 |
| Negative | 33 | 0.10±0.07 | 59 | 0.11±0.06 | 0.55 |
| Distant metastasis |  |  |  |  |  |
| Yes | 32 | 0.09±0.06 | 5 | 0.06±0.06 | 0.28 |
| No | 83 | 0.11±0.07 | 62 | 0.10±0.06 | 0.55 |
| CEA |  |  |  |  |  |
| Normal (< 3.4 µg/L) | 47 | 0.12± 0.07 | 38 | 0.11±0.06 | 0.27 |
| Elevated (> 3.4 mg/L) | 68 | 0.09±0.06 | 20 | 0.07± 0.05 | 0.08 |

**Table S2. The Tumour Regression Grade (TRG) (modification of Ryan et al.)**

| **Description** | **Tumour regression score** |
| --- | --- |
| No viable cancer cells (Complete response) | 0 |
| Single cells or rare small groups of cancer cells (Near-complete response) | 1 |
| Residual cancer with evident tumour regression but more than single cells or rare small groups of cancer cells (Partial response) | 2 |
| Extensive residual cancer with no tumour regression (Poor or no response) | 3 |

**Table S3. Characteristics of patients who received neoadjuvant therapy.**

| Characteristics | TRG 0-1 | TRG 2-3 |
| --- | --- | --- |
| Age, years | 52.00±10.55 | 53.88±10.63 |
| Sex |  | |
| Male | 8(61.54%) | 11(68.75%) |
| Female | 5(38.46%) | 5(31.25%) |
| Neoadjuvant therapy |  | |
| Chemotherapy | 11(84.6%) | 15(93.8%) |
| Immunotherapy | 2(15.4%) | 1(6.3%) |

**Table S4. Types of pathology and organs involved**

| **Characteristics** | **N** | **Pathologic diagnosis (n)** | **affected organ (n)** |
| --- | --- | --- | --- |
| BCD | 31 | Inflammatory Polyps (3)  Adenomas (28) | Colon (28)  Rectum (3) |
| BNC | 25 | inflammatory pseudotumour (2)  abscess (1)  Chronic inflammation with fibroplasia (21)  granuloma（1） | upper lobe of the right lung (5)  middle lobe of the right lung (6)  lower lobe of the right lung (4)  upper lobe of the left lung (8)  lower lobe of the left lung (2) |

BCD: Benign Colorectal Disease; BNC, Benign Nodule Controls.
