# Supplementary material for: Peripheral Blood T‐Cell Receptor Repertoire Diversity as a Potential Biomarker in the Diagnosis and Treatment Evaluation of Colorectal and Lung Cancers: A Prospective Observational Study
Source: Cancer Med. 2025 May 19;14(10):e70937. doi: 10.1002/cam4.70937 (PMC12086972; doi:10.1002/cam4.70937)
Supplement: Supplementary file 5 — Data S1. [file CAM4-14-e70937-s002.docx]

**SUPPLEMENTARY METHODS**

***RNA extraction using Trizol method***

Samples were centrifuged (Thermol Sorvall ST 40R with TX-1000 rotor Thermo Fisher Scientific, Waltham, MA, USA) at 3000 g for 10 min at ambient temperature. The supernatant (1.5 mL) was collected and transferred to a 5mL centrifuge tube, adding 300 μL of chloroform and mixed thoroughly. The mixture was allowed to rest at 25℃ for a duration of 5 minutes, followed by adding 500 μL of isopropanol and mixing well. Following a 10-minute incubation at ambient temperature, the sample was centrifuged at 12,000 g for 10 min at 4℃. The supernatant was then discarded. Subsequently, 1 mL of 75% ethanol was introduced to wash the RNA pellet. Following centrifugation at 7500 g for a period of 5 min at a temperature of 4℃, the supernatant was discarded, and the RNA pellet was left to dry at the ambient temperature for 5 min. Subsequently, the RNA pellet was reconstituted in 30μL of DEPC-treated water and stored at a temperature of -80℃.
